# Endometriosis in the time of internet: how web navigation affects women with endometriosis

**DOI:** 10.1080/07853890.2023.2215537

**Published:** 2023-05-30

**Authors:** Alessandro Arena, Eugenia Degli Esposti, Benedetta Orsini, Elisa Moro, Simona Del Forno, Laura Cocchi, Michele Altieri, Paolo Casadio, Renato Seracchioli

**Affiliations:** a Division of Gynaecology and Human Reproduction Physiopathology, IRCCS Azienda Ospedaliero-Universitaria di Bologna, Bologna, Italy; b Department of Medical and Surgical Sciences (DIMEC), University of Bologna, Bologna, Italy

**Keywords:** Endometriosis, anxiety disorders, Internet, web

## Abstract

**Objectives:**

Patients often search for health-related information on the internet allthough this trend may have some benefits, it also has some risks, such as misinformation. The aim of this study is to evaluate how Internet information seeking affect the level of anxiety in patients with endometriosis.

**Materials and methods:**

This prospective observational study was conducted at our outpatient clinic between March 2019 and December 2020. We enrolled We enrolled all patients with a confirmed sonographic diagnosis of endometriosis who had sought information about the disease prior to our visit. We divided them into two groups based on the source of information (Internet only vs multiple sources). Before the visit, we asked women to fill-in validated questionnaires about anxiety, such as the Generalized Anxiety Disorder-7 (GAD) and the Spielberg State Trait Anxiety Inventory (STAI- Y6) and the Endometriosis Health Profile (EHP) − 5. After the visit, the STAI-Y6 was resubmitted to each woman.

**Results:**

We enrolled 200 women who filled-in the questionnaires: 46 reported the Internet as the only source of information, 52 sought information also from medically qualified sources, 74 consulted only healthcare professionals, and 28 resorted to medical journals. Women who used the Internet as their exclusive source of information were younger on average and their STAI-Y6 score after the visit was significantly lower compared to other group (34.1 ± 11.5 vs 42.1 ± 14.7, *p* = .001). Moreover, the difference between the STAI-Y6 scores before and after our assessment was higher in these women (-18.3 ± 14.7 vs −10.3 ± 16.5, *p* = .003).

**Conclusions:**

Women who sought information online were younger, had lower levels of state trait anxiety after our medical evaluation, and a had a greater reduction in anxiety levels after our examination compared to women who consulted other sources to learn more about endometriosis.

**KEY MESSAGES**
- Women using only Internet are younger than those who use other sources of information.
- Women who researched symptoms online showed higher pre-examination anxiety levels.
- Patients with severe pain symptoms consulted both the internet and professionals.

## Introduction

Endometriosis is one of the leading causes of pelvic pain, affecting up to 15% of reproductive-aged women [1]. Not only women suffering from this disease are afflicted by painful symptoms [2,3], but they also have psychological issues, most notably higher anxiety and distress levels, and greater depression rates compared to healthy controls [4–6].

Women affected by endometriosis show a negative correlation between pain and general health, intended as mental, physical and emotional wellbeing [7]. In addition to the undeniable negative correlation between chronic pain and quality of life (QoL), many additional factors must be taken into account, including poor sex life, diagnostic delay and uncertainty [8], minimization of their symptoms by clinicians [9], infertility [10], and impaired education and work life [11].

Nowadays, another important factor should be considered: the impact of the Internet and online self-diagnosis before a professional evaluation. The Internet has become an irreplaceable part of everyday life, and a growing number of people use the web as a source of information for health conditions [12,13]. On one hand, this tendency may have potential benefits, educating patients about specific health conditions and empowering them in their health decision-making process [14]. However, some concerns have been raised regarding the high risk of misinformation and the effects of this trend on the relationship between patients and clinicians. Given the high prevalence of anxiety among women suffering from endometriosis, it is essential to have better insights into the impact that online research has on this subset of patients. The purpose of our study is to assess how internet information-seeking affects patients’ anxiety levels before and after outpatient medical examination. Moreover, we aimed to understand which patients’ characteristics represent risk factors for the propensity to use the Internet as the only source of information.

## Materials and methods

This prospective observational study was conducted at our referral center for the diagnosis and treatment of endometriosis in Bologna, between March 2019 and December 2020. The study population included all women referred to our evaluation with clinical or sonographic suspicion of endometriosis. Informed consent was obtained from all participants included in the study. The study protocol was approved by the local Ethics Committee (201/2017/O/Sper).

We excluded patients younger than 18, with poor knowledge of the Italian language, currently pregnant or menopausal, with a personal history of malignancies or previous surgery for endometriosis, with concurrent non-gynecological causes of pelvic pain, and diagnosed with psychiatric conditions according to the Diagnostic and Statistical Manual of Mental Disorders (DSM-5).

Before our visit, all women compiled a questionnaire analyzing socio-demographic information such as age, relationship status, educational level, and pre-existing anxiety levels. Women were asked whether they had tried to learn more about endometriosis before our visit and to list their sources of information. We excluded from the analysis women who did not search for information about their condition before our assessment. The study population was divided into two groups: patients who only sought information online and patients who resorted to other qualified sources, such as general practitioners, gynecologists, and medical/scientific literature.

Anxiety was evaluated using two validated questionnaires: the Italian version of Generalized Anxiety Disorder (GAD) − 7 [15] and the Spielberg State Trait Anxiety Inventory (STAI) Y6 [16]. The GAD-7 is a seven-item questionnaire employed to determine the status of generalized anxiety. The STAI-Y6 is a frequently used measure of state anxiety (the anxiety patients presently feel).

We asked all women to rate the severity of their endometriosis-related symptoms (dysmenorrhea, dysuria, dyschezia, dyspareunia, and chronic pelvic pain) using the Numerical Rating Scale (NRS) and the 11-item questionnaire of Endometriosis Health Profile (EHP) − 5 [17].

After completing the surveys, all women underwent a thorough gynaecological evaluation by a senior consultant, highly trained in endometriosis, as well as a transvaginal and transabdominal ultrasound. After the visit, the STAI-Y6 was resubmitted to each participant to evaluate changes in anxiety levels after the medical consult. Doctors were blinded to the results of pre-and post-visit questionnaires.

### Statistical analysis

This investigation was conceived as a pilot study; therefore, we did not perform a power analysis. During the study period, we enrolled all consecutive patients referred to our centre for a second-level evaluation due to a previous clinical suspicion or imaging diagnosis of endometriosis.

Results were expressed as mean ± SD for normally distributed variables, or as numbers and percentages for categorical variables. The normal distribution of continuous variables was assessed using the Kolmogorov-Smirnov test. We compared the characteristics of patients by chi-square or Fisher’s exact tests for categorical variables. Unpaired Student’s t-test and Mann–Whitney test were used respectively to compare continuous parametric and non-parametric variables between the two groups, whereas one-way ANOVA was used to compare continuous variables between three or more groups. A *P*-value of <0.05 was considered significant for all tests.

All statistical analyses were performed using the Statistical Package for the Social Sciences software (IBM SPSS v. 25, SPSS Inc.).

## Results

Between March 2019 and December 2020, 234 women were referred to our centre for a second evaluation due to a previous clinical suspicion or imaging diagnosis of endometriosis. Thirty-four women were ruled out from the analysis based on the exclusion criteria.

Two hundred women who had conducted specific research about endometriosis before our visit completed the questionnaires and listed their sources of information. Among them, 46 women reported the Internet as the single source of knowledge about their condition, while 154 women resorted to other sources. Particularly, 52 women obtained information both from the Internet and healthcare providers, such as general practitioners or gynaecologists, 74 patients had consulted only medical professionals, and 28 declared they had only consulted scientific papers or medical journals.

Anamnestic characteristics of patients at baseline and results of the questionnaires about anxiety are summarized in Table 1. Women who only sought information online were significantly younger than those who consulted other sources and they had lower post-evaluation STAI-Y6 scores compared to their counterparts (34.1 ± 11.5 vs 42.1 ± 14.7, *p* = .001). Additionally, the difference between the STAI-Y6 scores before and after our examination was significantly higher in these women (−18.3 ± 14.7 vs −10.3 ± 16.5, *p* = .003). Most non-Italian women reported the Internet as their sole source of information, as opposed to Italian women, who mainly consulted other sources (28.3% vs 13.6%, and 71.7% vs 86.4% respectively, *p* = .02). No differences were observed between the two groups in terms of the global scores of the EHP-5, as detailed in Table 2. However, regarding the specific items of this questionnaire, our data showed that women who only used the Internet as their source of information were significantly more worried about the difficulties in obtaining pregnancy (2.7 ± 1.2 vs 1.9 ± 1.4, *p* <.001). On the other hand, women who had consulted other sources were mostly concerned about the possibility that therapy might be unsuccessful and about their inability to perform their duties at work (1.8 ± 1.4 vs 1.3 ± 1.2, *p* = .02.; 1.7 ± 1.3 vs 1.0 ± 1.2, *p* = .005). Also, women who reported only the Internet as their source of information were less likely to be already on medical therapy for endometriosis (Table 2).

**Table 1.** Baseline characteristics of the study population.

Characteristic	Only internet *N* = 46 (%)	Other sources *N* = 154 (%)	*p* Value
**Age (y)**	31.4 ± 7.3	34.6 ± 7.6	**.01**
**Marital status**			.68
Never married	19 (41.3%)	73 (47.4%)	
Married/civil partnership	24 (52.2%)	76 (49.4%)	
Divorced	2 (4.3%)	4 (2.6%)	
Widowed	1 (2.2%)	1 (0.6%)	
**Level of education**			.34
Primary school	1 (2.2%)	11 (7.1%)	
Middle school diploma	4 (8.7%)	20 (13.0%)	
High school diploma	21 (45.7%)	62 (40.3%)	
Academic degree	19 (41.3%)	50 (32.5%)	
Other	1 (2.2%)	11 (7.1%)	
**Birthplace**			**.02**
Italy	33 (71.7%)	133 (86.4%)	
Other Countries	13 (28.3%)	21 (13.6%)	
**GAD-7 scoring**			**.03**
Minimal (0-4)	6 (13.0%)	18 (11.7%)	
Mild (5-9)	16 (34.8%)	68 (44.2%)	
Moderate (10-14)	16 (34.8%)	24 (15.6%)	
Severe (15-21)	8 (17.4%)	44 (28.6%)	
**Pre-visit STAI-Y6**	52.3 ± 11.5	52.3 ± 15.3	.99
**Post-visit STAI-Y6**	34.1 ± 11.5	42.1 ± 14.7	**.001**
**Delta STAI-Y6**	−18.3 ± 14.7	−10.3 ± 16.5	**.003**

Values are expressed as mean ± standard deviation or counts (percentages).

Bold indicates statistical significant values (*p* < 0.05).

**Table 2.** Symptoms and medical conditions assessed during medical examination.

Characteristic	Only internet *N* = 46	Other sources *N* = 154	*p* Value
**EHP-5 score**	39.7 ± 21.3	42.8 ± 22.8	.41
1. Found it difficult to walk because of the pain	1.3 ± 0.9	1.5 ± 1.3	.27
2. Felt as though symptoms are ruling your life?	1.8 ± 1.4	1.9 ± 1.2	.44
3. Had mood swings?	2.4 ± 1.1	2.5 ± 1.1	.45
4. Felt others do not understand what you are going through?	1.7 ± 1.5	2.1 ± 1.4	.11
5. Felt your appearance has been affected?	1.7 ± 1.2	1.7 ± 1.4	.98
6. Been unable to carry out duties at work because of the pain?	1.0 ± 1.2	1.7 ± 1.3	**.005**
7. Found it difficult to look after your child/children?	1.2 ± 1.3	1.1 ± 1.2	.43
8. Felt worried about having intercourse because of the pain?	1.5 ± 1.3	1.6 ± 1.4	.53
9. Felt doctor(s) think it is all in mind?	0.8 ± 1.2	1.0 ± 1.3	.46
10. Felt frustrated because treatment is not working?	1.3 ± 1.2	1.8 ± 1.4	**.02**
11. Felt depressed at the possibility of not having children/more children?	2.7 ± 1.2	1.9 ± 1.4	**<.001**
**Symptoms**			
Dysmenorrhea (VAS)	5.8 ± 3.1	6.6 ± 2.7	.09
Chronic pelvic pain (VAS)	4.4 ± 3.2	4.8 ± 3.4	.56
Dyspareunia (VAS)	4.1 ± 3.2	4.3 ± 3.3	.71
Dyschezia (VAS)	2.3 ± 3.1	2.6 ± 2.9	.57
Dysuria (VAS)	1.5 ± 2.3	1.9 ± 2.9	.37
**Already on medical therapy**	14 (30.4%)	74 (48.1%)	**.03**
**Medical therapy prescribed after examination**	20 (71.4%)	66 (76.7%)	.57

Values are counts (percentages) or mean ± standard deviation.

Bold indicates statistical significant values (*p* < 0.05).

The subsequent analysis of the four subgroups confirmed our previous evaluations, as shown in supplementary material. Educational levels and marital status did not seem to influence the chosen source of information (Supplementary Material).

Moreover, we observed a peculiar trend for chronic pelvic pain, dyspareunia, dyschezia and dysuria: patients who only sought information online or on medical journals had significantly less severe symptoms compared to women who resorted to both medical advice and the Web (Supplementary Material). The same trend was noted for the EHP-5 and the STAI-Y6 scores. Also, the mean difference between the pre-visit and post-visit STAI-Y6 scores (Delta STAI-Y6) was more pronounced for those women who only researched the Web than for patients who also listened to healthcare professionals prior to our evaluation (−18.3 ± 14.7 vs −7.3 ± 17.8, *p* = .002).

Regarding the GAD-7, the ANOVA analysis showed that the relative majority of patients with severe trait anxiety (scoring between 15 and 21) had sought information both online and from healthcare professionals, as opposed to only two patients with minimal anxiety who had resorted to multiple sources of information (38.5% vs 8.3%, *p* = .006).

## Discussion

Endometriosis is a pervasive disease, impairing different and multiple aspects of patients’ life. Not only women suffering from this disease are afflicted by painful symptoms, but they often have psychological issues, most notably higher anxiety and distress levels, and greater depression rates compared to healthy controls [7,18,19]. Considering the complexity of the condition and the fact that up to 70% of adults rely on the Internet to search for health information, it is important to clarify the role of online health-related information search [20]. The knowledge obtained by surfing the internet can empower patients and can redefine the patient’s position concerning their condition, letting them exercise more control over their disease [14].

This is the first study to investigate how online information-seeking can affect anxiety levels in women with endometriosis, and which patients’ characteristics are related to the propensity to use the Internet as the only source of information.

Women who only resort to the Internet are markedly younger, confirming a trend already observed in the literature [21]. These results are not surprising considering that younger generations are more familiar with new information and communication technologies and the Web. Patients can appreciate the opportunity to privately investigate difficult and embarrassing topics and to exert greater control over the rate at which they learn new medical information, reducing the “information overload” sensation [22]. In line with this, our data showed that women who searched for information only through the Internet were mostly concerned about infertility and experienced depressive symptoms more frequently when dealing with this topic. Infertility has become a hot topic on the Internet, and a systematic review showed that infertile women tend to access the Internet to satisfy their information needs, and to find psychological support and medical assistance [23]. These data were confirmed by a retrospective analysis that found that most posts related to minimally invasive gynaecological surgery on Instagram were about endometriosis and infertility [24].

Another important aspect to consider is to provide patients with adequate information regarding the correlation between endometriosis and cancer risk, another topic that patients frequently run into while surfing online. It is important to remember that data are showing that patients with endometriosis have a higher risk of developing ovarian cancer in case of atypical endometriosis that persists into old age. However, women should be informed that the cause-effect relationship of this association is still unclear, as is the quantification of the absolute risk of malignant degeneration [25].

Women with higher levels of generalized anxiety (GAD-7 scoring between 15 and 21) usually sought medical information both on the internet and by consulting medical professionals. Similarly, patients with more severe pain symptoms consulted both the Internet and healthcare providers when looking for information about their condition (Supplementary Material S1). We can conclude that more intense pain symptoms and higher anxiety levels lead women to seek information through several different sources, possibly worsening the situation with misinformation and unresolved doubts.

The danger of misinformation is particularly relevant in this population, as our group has already demonstrated in a previous study [26]. Given the prominence that endometriosis has gained in recent years, a large number of pages, sites and blogs have been created to exchange medical advice about it, but the accuracy of this kind of information is questionable at best, or even openly faulty in some instances. Furthermore, up to 40% of people who search the Internet for medical news are less likely to believe a healthcare professional’s opinion if it is in contrast with what found on the Web [27].

Interestingly, we noted a relevant decrease in the STAI-Y6 score before and after our evaluation in patients who used only Internet as source of information. Furthermore, women who researched their symptoms and signs online presented very high pre-examination anxiety levels and showed a higher decrease in anxiety after a detailed interview with an expert gynaecologist, confirming our previous findings [28]. The medical examination and the outpatient interview allow the patient to obtain detailed information about her disease and resolve important doubts about the need for therapy, the indications for surgery, and the possible complications related to it. In this setting, it is possible to clarify the rationale for medical and surgical treatment, also considering each patient’s expectations regarding the efficacy of the treatment on pain symptoms, the desire for pregnancy, and the extent of the pathology [29].

Women who had already started medical therapy for endometriosis were less likely to search the Internet for health information, compared to naïve patients. We may infer that these women had already had a medical consultation when hormonal therapy was first prescribed and consequently had no particular need to search the Web for additional information about their condition.

On the other hand, non-native Italian-speaking patients showed a decided tendency to resort to the Internet to obtain medical information, as opposed to Italian patients. We can hypothesize that the Internet offers these patients an easier access to information in their mother tongue, improving their comprehension of the disease.

### Limitations

Despite its novelty, this study presents certain limitations. Firstly, this work reflects the experience of a single centre and due to the small sample size of the study, our results cannot be generalized.

The fact that the women were visited by different gynaecologists is an important limitation, despite the standardised modus operandi of our centre. Furthermore, we only submitted questionnaires to patients with an optimal comprehension of the Italian language, further limiting the generalisability of our results. We did not investigate the quality of the information searched online by the women and the possible misinformation which could have impacted their anxiety levels. Another limitation is the possibility of overlapping diseases, such as fibromyalgia and irritable bowel syndrome (IBS), which may cause chronic painful symptoms. Despite excluding patients with this kind of diagnosis, some patients might not have been aware of these conditions, determining a bias in our analysis.

## Conclusions

In light of our findings, gynaecologists evaluating women with suspected endometriosis for the first time should be aware that they are dealing with highly anxious patients, who probably have searched the Internet extensively for their long-unanswered doubts. Physicians conducting first outpatient evaluations should know that this is a highly stressful moment for these patients, one that could make a difference in terms of subsequent anxiety reduction. A thorough and tailored interview by trained and competent doctors is essential to reduce women’s discomfort, as our data show. Doctors should also keep in mind that young and foreign patients often use the Internet as their only source of health information and that infertility is a major concern for these women.

It is essential to remember that young women with endometriosis may have a better understanding of their condition than before, and a deeper knowledge, and consequently may express a desire to become personally involved in their diagnostic and therapeutic management and to stop being passive recipients of one-sided, top-down decision-making processes. Clinicians should cultivate this tendency to their advantage to build stronger bonds with these patients, helping them overcome not only their physical symptoms but also lightening their psychological burden. In this scenario, the Internet should be seen by clinicians as a unique opportunity to provide correct medical information in an easily understandable and non-paternalistic language to reach a great number of patients.

## Supplementary Material

Supplemental Material

## Data Availability

The data that support the findings of this study are available from the corresponding author, [B.O.], upon reasonable request.
