# Supplementary material for: Endometriosis in the time of internet: how web navigation affects women with endometriosis
Source: Ann Med. 2023 May 30;55(1):2215537. doi: 10.1080/07853890.2023.2215537 (PMC10234130; doi:10.1080/07853890.2023.2215537)
Supplement: Supplemental Material [file IANN_A_2215537_SM2775.docx]

**Supplementary Material S1.** Baseline characteristics and examination findings based on the source of information. Values are expressed as counts (percentages) or mean ± standard deviation.

| Characteristic | Medical Journals  N = 28 | Only healthcare professionals  N = 74 | Only Internet  N = 46 | Healthcare professionals and Internet  N = 52 | p |
| --- | --- | --- | --- | --- | --- |
| **Birthplace** |  |  |  |  | **.002** |
| Italy (n = 166) | 18 (10.8%) | 66 (39.8%) | 34 (20.5%) | 48 (28.9%) |  |
| Other Country (n = 34) | 10 (29.4%) | 8 (23.5%) | 12 (35.3%) | 4 (11.8%) |  |
| **Marital Status** |  |  |  |  | .47 |
| Never married (n = 92) | 9 (9.8%) | 38 (41.3%) | 19 (20.7%) | 26 (28.3%) |  |
| Married / civil partnership (n = 100) | 18 (18.0%) | 34 (34.0%) | 24 (24.0%) | 24 (24.0%) |  |
| Divorced (n = 6) | 0 (0.0%) | 2 (33.3%) | 2 (33.3%) | 2 (33.3%) |  |
| Widowed (n = 2) | 1 (50.0%) | 0 (0.0%) | 1(50.0%) | 0 (50.0%) |  |
| **Level of education** |  |  |  |  | .25 |
| Primary school (n = 12) | 4 (33.3%) | 4 (33.3%) | 1 (8.3%) | 3 (25.0%) |  |
| Middle school diploma (n = 24) | 5 (20.8%) | 7 (29.2%) | 4 (16.7%) | 8 (33.3%) |  |
| High school diploma (n = 8) | 10 (12.0%) | 30 (36.1%) | 21 (45.7%) | 22 (26.5%) |  |
| Academic degree (n = 69) | 5 (7.2%) | 29 (42.0%) | 19 (27.5%9 | 16 (23.2%) |  |
| Other (n = 12) | 4 (33.3%) | 4 (33.3%) | 1 (8.3%) | 3 (25.0%) |  |
| **GAD-7 scoring** |  |  |  |  | **.006** |
| Minimal (0-4) (n = 24) | 4 (16.7%) | 12 (50.0%) | 6 (25.0%) | 2 (8.3%) |  |
| Mild (5-9) (n = 84) | 12 (14.3%) | 38 (45.2%) | 16 (34.8%9 | 18 (34.6%) |  |
| Moderate (10-14) (n = 40) | 2 (5.0%) | 10 (25.0%) | 16 (40.0%) | 12 (30.0%) |  |
| Severe (15-21) (n = 52) | 10 (19.2%) | 14 (26.9%) | 8 (15.4%) | 20 (38.5%) |  |
| **Already on medical therapy**  **(n = 88)** | 6 (6.8%) | 34 (38.6%) | 14 (15.9%) | 34 (38.6%) | **<.001** |
| **Medical therapy prescribed after examination.**  **(n= 86)** | 18 (20.9%) | 36 (41.9%) | 20 (23.3%) | 12 (14.0%) | .64 |
| **Age (y)** | 31.6 ± 8.4 | 35.4 ± 7.4 | 31.4 ± 7.3 | 34.9 ±7.2 | **.01** |
| **Symptoms** |  |  |  |  |  |
| Dysmenorrhea (VAS) | 6.8 ± 2.5 | 6.5 ±2.7 | 5.8 ± 3.1 | 6.5 ± 2.9 | .41 |
| Chronic pelvic pain (VAS | 3.9 ± 3.7 | 4.2 ± 3.3 | 4.4 ± 3.2 | 6.0 ± 2.9 | **.007** |
| Dyspareunia (VAS) | 4.0 ± 3.6 | 4.0 ± 3.4 | 4.1 ± 3.2 | 5.0 ± 3.0 | .35 |
| Dyschezia (VAS) | 2.0 ± 1.6 | 2.5 ± 3.1 | 2.3 ± 3.1 | 3.0 ± 3.1 | .51 |
| Dysuria (VAS) | 0.9 ± 1.8 | 1.7 ± 2.7 | 1.5 ± 2.3 | 2.7 ± 3.4 | **.02** |
| **EHP-5** | 29.9 ± 18.7 | 40.4 ± 20.0 | 39.7 ± 21.2 | 53.3 ± 24.2 | **<.001** |
| Been unable to carry out duties at work because of the pain? | 1.2 ± 1.2 | 1.5 ± 1.3 | 1.0 ± 1.2 | 2.0 ± 1.3 | **.001** |
| Felt frustrated because treatment is not working? | 1.4 ± 1.3 | 1.6 ± 1.2 | 1.3 ± 1.2 | 2.5 ± 1.4 | **<.001** |
| Felt depressed at the possibility of not having children/more children? | 1.3 ± 1.2 | 2.0 ± 1.4 | 2.7 ± 1.2 | 2.0 ± 1.5 | **<.001** |
